# Latent Profile Analysis of Distinct Patterns of Treatment and Assessment Practices among Youth Mental Health Providers

**DOI:** 10.1007/s10488-026-01498-7

**Published:** 2026-03-27

**Authors:** Evelyn Cho, Kristin M. Hawley

**Affiliations:** 1 University of Oregon, Portland, United States; 2 University of Missouri, Columbia, United States

**Keywords:** Evidence-based assessment, Evidence-based practice, Evidence-based treatment, Youth mental health, Usual care

## Abstract

Evaluations of youth mental health providers’ treatment and assessment practices have largely examined trends in evidence-based treatment (EBT) and standardized measures use separately. Evidence-based practice (EBP) integrates EBTs with accurate, valid assessment. Thus, there is a need to understand how individual providers integrate treatment and assessment practices in routine care. To address this gap, the current study used latent profile analysis to derive distinct profiles of assessment and treatment practices, identified provider and practice settings characteristics that predict profiles, and compared provider-reported top influences on their current practices between profiles. In a multidisciplinary national survey, 1,092 youth mental health providers self-reported their assessment (i.e., use of standardized, unstandardized assessment measures) and treatment (i.e., strategies common across EBTs for multiple problem areas, strategic specific to EBTs for a single problem area, and strategies not included in EBTs for any of the problem areas) practices. Four distinct profiles were identified. The largest profile, “EBT Eclectics” (73%), reported a mix of common and specific EBT and other treatment strategies and unstandardized measures, but minimal standardized measures use. The profile that most closely resembled EBP, “EBP Integrationists” (17%), reported frequent use of common and specific EBT strategies alongside some standardized measures use. The two smaller profiles were differentiated by their relative use of specific EBT (“Key Ingredients Specialists”, 6%) and other treatment strategies (“Generalists”, 5%). Profiles were differentiated by provider and practice characteristics, and provider-reported top influences. Findings highlight future directions to enhance EBP in routine care settings.

## Introduction

Evidence-based treatments (EBTs) for common youth mental health concerns have demonstrated clinical benefit in over fifty years of youth psychotherapy research (Weisz et al., 2017). These EBTs, however, remain underutilized in routine youth mental health care settings (Becker et al., 2013; Bruns et al., 2016). This research-to-practice gap has galvanized workforce development efforts to train providers in EBTs (e.g., Leffler et al., 2013; Parrish & Rubin, 2011) and publicly-funded initiatives to integrate EBTs into routine care settings (Cho et al., 2022; McHugh & Barlow, 2010). Rigorous evaluation of EBT use is needed to gauge the success of these implementation efforts, identify continued EBT implementation needs, and guide future efforts to optimize youths’ access to EBTs in routine care (Cho et al., 2022; Proctor et al., 2011).

To date, evaluation of EBT use has primarily focused on overall trends describing whether and to what extent youth mental health providers use EBTs in routine care settings. Early surveys of EBT use asked providers about their use of “evidence-based practices” (Nelson & Steele, 2007), use of specific EBTs (DiMeo et al., 2012; Jensen-Doss et al., 2009; Walrath et al., 2006), or treatment manuals broadly (Addis & Krasnow, 2000; Becker et al., 2013). These studies showed that while providers generally reported using “EBP” (Nelson & Steele, 2007), only a minority consistently use specific EBT manuals or manualized treatments (Becker et al., 2013; Jensen-Doss et al., 2009; Walrath et al., 2006), even when substantial infrastructure exists to support their use (Jensen-Doss et al., 2009), and with most making adaptations to EBTs (DiMeo et al., 2012). More recently, given considerable overlap in prescribed treatment components across EBTs for similar problems (e.g., behavioral parent training strategies for disruptive behavior disorders; Garland et al., 2008) and across problem areas (Chorpita et al., 2005), evaluations have honed in on providers’ use of these EBT elements. Provider self-report studies suggest that providers regularly incorporate common EBT elements into routine practice (Cho et al., 2019; Higa-McMillan et al., 2017). However, observational studies indicate that providers deliver these EBT components with low frequency and intensity (Brookman-Frazee et al., 2021; Garland et al., 2010). Together, these findings suggest that while providers do not regularly use EBT manuals, they integrate components of these EBTs into routine practice, though with limited extensiveness.

Several studies highlight the need to move beyond whether and to what extent EBTs are used in routine care settings to unpacking *how* they are delivered. One guiding framework is evidence-based practice (EBP), defined as “the integration of the best available research with clinical expertise in the context of patient characteristics, culture, and preferences” (American Psychological Association, 2006). In the EBP decision-making framework, treatment delivery is guided, in part, by the best available research evidence including, but not limited to, randomized clinical trials (American Psychological Association, 2006; Roberts et al., 2017; Spring, 2007). These RCTs have consistently demonstrated support for EBTs for common youth mental health concerns, making treatment delivery consistent with clinical trials treatment delivery a key consideration in EBP. Specifically, in these clinical trials, EBT delivery maximizes adherence to prescribed treatment components while minimizing proscribed components (Perepletchikova et al., 2007). Adherence to prescribed EBT components is considered a necessary prerequisite to clinical outcomes (Proctor et al., 2011) and has been linked to treatment outcomes in some prior studies (e.g., Collyer et al., 2020; Huey et al., 2000). In routine care settings, however, a substantial number of providers report an eclectic orientation (Becker et al., 2013; Beidas et al., 2015; Garland et al., 2010), and provider self-report and observational coding of treatment sessions show that providers mix EBTs with other treatment strategies not commonly prescribed in EBTs (e.g., using play or art therapy, identifying youth strengths, insight building; Cho et al., 2019, 2022; Garland et al., 2010; Higa-McMillan et al., 2017). A closer look within EBT strategies suggests that providers may disproportionately omit the “key ingredients” of EBTs (Cho et al., 2019, 2022; Higa-McMillan et al., 2017). For example, among providers treating youths with anxiety within Hawaii’s state-funded mental health system, only 15% of cases received exposure which is prescribed in 86% of EBTs, while 0–90% of youths received treatment strategies not prescribed in any of the EBTs (Higa-McMillan et al., 2017). These findings suggest that on average providers use a range of treatment strategies, including those inconsistent with the best available research. However, this prior work evaluated EBT components use *across* providers. It remains unclear whether the mix of treatment strategies used by individual providers reflects those prescribed and emphasized in EBTs. One recent study used latent profile analysis (LPA), a person-centered quantitative approach, to begin addressing this gap (Becker-Haimes et al., 2019). Based on youth-serving providers’ self-reported use of cognitive, behavioral, family, and psychodynamic treatment strategies for a representative case, the LPA identified four treatment practice profiles including clinicians whose practices reflected primarily family-based treatment strategies, and those who reported low, moderate, and high use of strategies from all four treatment families. Building on this work, person-centered approaches can be applied to treatment strategies with and without research support to explore variations in the mix of treatment strategies used by individual providers.

A second tenet of EBP with implications for EBT delivery is providers’ clinical expertise. Clinical expertise within EBP involves multiple competencies critical to positive client outcomes (American Psychological Association, 2006). Of these competencies, those involving accurate assessment (e.g., diagnostic judgment, and client progress monitoring) to guide treatment decisions are key to EBT implementation. It is recommended that standardized, validated assessment directs the selection of EBTs at the start of treatment, guides decisions throughout treatment, and informs the termination of care (Hunsley & Mash, 2007). Unfortunately, survey studies and health records data consistently show that on average standardized measures use is infrequent among providers in routine care settings (Cashel 2002; Cho et al. 2021; Garland et al. 2003; Gilbody et al. 2002; Hatfield and Ogles 2004; Ionita and Fitzpatrick 2014; Jensen-Doss et al. 2018a, b; Morris et al. 2021). A complement to these variable-centered studies, a latent profile analysis of providers’ self-reported assessment practices in a national sample of youth mental health providers identified four profiles of providers’ standardized and unstandardized measures use (Cook et al., 2017). Three profiles represented providers who, consistent with prior research, infrequently used standardized measures, with “unstandardized assessors” (77%) who heavily favored use of unstandardized measures over standardized measures, “broad-spectrum assessors” (12%) who used both standardized and unstandardized measures, and “minimal assessors” (6%) who reported infrequent use of all measures with the exception of unstructured clinical interviews and informal mental status exams. Only one profile representing 12% of providers used standardized measures “sometimes” to “often.” This study highlighted variations in providers’ assessment practices, with some providers using standardized measures more often than suggested by variable-centered approaches. It remains unclear, however, whether providers who use standardized measures integrate these assessment tools with EBTs.

To address these two knowledge gaps, the current study aimed to derive distinct patterns of providers’ assessment and treatment practices for youths with common mental health concerns in routine care settings. Using a national, multidisciplinary survey of youth mental health providers (Cho et al., 2019), first, we aimed to use LPA to identify *profiles* reflecting distinct assessment and treatment strategy patterns for common youth mental health problems (i.e., anxiety, depression, disruptive behavior). We had no a priori hypotheses on the number or types of practice patterns that would emerge. Once profiles are derived, understanding the factors that distinguish these profiles could inform future efforts to tailor implementation strategies based on providers’ practice patterns. Therefore, our second aim was to identify provider and practice setting characteristics that predicted profile membership. Based on prior research largely using variable-centered approaches, we hypothesized that the following factors would differentiate practice profiles: provider characteristics including psychology discipline (Becker-Haimes et al., 2019; Higa-McMillan et al., 2015), doctoral degree (Becker-Haimes et al., 2019; Campbell et al., 2013; Cook et al., 2017; Higa-McMillan et al., 2015), time since degree (Brookman-Frazee et al., 2010; Cho et al., 2019), learning theory orientation (Becker et al., 2013; Becker-Haimes et al., 2019; Nelson & Steele, 2007), attitudes toward research evidence (Beidas et al., 2017; Cho et al., 2019; Nelson & Steele, 2007; Okamura et al., 2019), attitudes toward standardized assessment (Cho et al., 2023; Cook et al., 2017; Garland et al., 2003; Hatfield & Ogles, 2004; Jensen-Doss et al., 2024; Jensen-Doss & Hawley, 2010; Jensen-Doss & Hawley, 2011); and practice setting characteristics including private practice (Jensen-Doss et al. 2018a, b), opportunities for supervision and consultation (Beidas & Kendall, 2010; Benjamin Wolk et al., 2016; Nelson & Steele, 2007), proportion of low-income and racial and ethnic minority cases in their caseload (Brookman-Frazee et al., 2010; Cho et al., 2019; Higa-McMillan et al., 2015), and client comorbidity (Addis et al., 1999; Nelson et al., 2006). Our third aim was to describe provider-reported top influences on their current practices and test whether these influences differed across profiles.

## Method

### Procedures

The Tailored Design Method (Dillman et al., 2009) was used to developed a survey that assessed (1) provider demographic and professional characteristics, (2) provider attitudes and perceptions about their practice settings, (3) assessment practices, and (4) treatment practices. Providers were randomly assigned to one of three survey versions that differed only in their instructions to providers to report their treatment strategies use with a recent (i.e., within the past year), representative case of youth anxiety, depression, or disruptive behavior. A total 5,000 (1,000 per guild) potential participants were identified from the membership rosters of the American Academy of Child and Adolescent Psychiatrists (AACAP), American Counseling Association (ACA), American Psychological Association (APA), Association of Marriage and Family Therapists (AMFT), and the National Association of Social Workers (NASW). Providers were mailed up to five times, resulting in a response rate of 61.53% (*n* = 2,863) after adjusting for 347 undeliverable surveys. The current study includes 1,092 providers who reported providing both assessment and intervention services to youths for common mental health concerns within the past year. All procedures were approved by the university’s Institutional Review Board. Full details of the survey methodology are provided in prior reports (Cho et al., 2019).

### Participants

Providers were 62.64% female; 92.22% White, 2.01% African American, 1.92% Asian American, 2.93% Latino/Hispanic, 0.92% other race or ethnicity, 0.64% Native American or American Indian; and were on average 52.58 years old (*SD* = 9.81). Most providers had doctoral degrees (53.57%), with an average 20.19 (*SD* = 10.16) years since their highest degree, and represented diverse disciplines (33.15% psychology, 24.27% counseling, 21.97% social work, 22.16% marriage and family therapy, 16.12% psychiatry, 3.85% other); with 39.93% reporting using a learning theory orientation with their recent, representative case. Providers’ practice settings included private individual or group practice (63.55%); outpatient clinics or community mental health clinics (18.86%); college, university, medical, or professional schools (9.25%); primary or secondary schools (9.07%); inpatient hospital or medical clinics (5.40%); residential treatment facilities or group homes (4.49%); day treatment facility or partial day hospitals (1.83%); HMO, PPO, or other managed care organizations (1.56%); and other settings (13.92%); with 51.74% reporting working solely in individual private practice. Providers’ case mix characteristics included on average 34.05% (*SD* = 32.07%) low-income youths and 31.18% (*SD* = 26.83%) racial and ethnic minority youths, and their recent, representative cases had on average 2.02 (*SD* = 1.29) co-occurring problems.

### Measures

#### Provider and Practice Setting Characteristics

Providers reported professional discipline (i.e., counseling, marriage and family therapy, psychiatry, psychology, social work); highest degree and date of this degree; and whether they worked solely in individual private practice. Providers reported case mix characteristics including the percentage of cases from low-income and racial and ethnic minority backgrounds. Providers selected the top three influences on their current practice from a list including graduate school coursework; continuing education; professional books or journals; insurance coverage rules; practicum, internship, residency; talking with colleagues; treatment manuals or practice guidelines; or other influences.

#### Provider Attitudes Toward Innovation and Evidence (PATIE; Cho et al., 2019)

The provider survey included items adapted from the Evidence-Based Practice Attitudes Scale (Aarons, 2004). In a prior study using these data, three of these items were indicators of a latent variable assessing attitudes toward innovation and evidence that predicted EBT use: “I enjoy learning new practices to help children and their families,” “I am willing to try new practice strategies even if they are very different from what I normally do,” “I like to try new types of practices that have been supported by research” (citation omitted). Items were rated on a 1 (“strongly disagree”) to 5 (“strongly agree”) scale, with higher ratings indicating more positive attitudes. Internal consistency was α=0.64; while alpha was below the cutoff of α=0.70 for adequate reliability, this variable was retained given that alpha increases with the number of items (Cronbach, 1951). In the current study, items were averaged to create a PATIE score.

#### Opportunities for Supervision and Consultation (OSC)

The provider survey included items assessing organizational culture and climate adapted from the Children’s Services Survey (Glisson, 2002). The authors identified four items that measured providers’ perceptions of the opportunities to receive supervision and consultation: “I regularly talk to colleagues about new practices relevant to my work,” “I would like to spend more time on ongoing training and consultation, but it is not possible” (reverse coded), “I have little opportunity for ongoing clinical supervision or consultation” (reverse coded), and “I feel comfortable with the amount of professional consultation and supervision I receive.” Items were rated on a 1 (“strongly disagree”) to 5 (“strongly agree”) scale, with higher ratings indicating more opportunities. Items were averaged to create a OSC score. The OSC was developed for this study and its psychometric properties have not been established. Internal consistency was α = 0.65.

Attitudes Toward Standardized Assessment (ASA; Jensen-Doss & Hawley, 2010).

The ASA is a 22-item measure of providers’ attitudes toward standardized assessment tools comprised of three subscales: (1) Benefit Over Clinical Judgment (*n* = 5 items) assessing perceptions that standardized measures have clinical utility beyond clinical judgment; (2) Psychometric Quality (*n* = 7) assessing perceptions about the psychometric properties of existing standardized measures; and (3) Practicality (*n* = 10) assessing perceptions about the practicality and feasibility of existing standardized measures. Items are rated on a 1 (“strongly disagree”) to 5 (“strongly agree”) scale, with higher ratings indicating more positive attitudes (several items are reverse coded). Internal consistency for the subscales was adequate, α = 0.72–0.75.

#### Assessment Practices

Providers rated how often they use standardized and unstandardized assessment practices with youths 3–17 years old on a 1 (“never or almost never”) to 5 (“all or most of the time”) scale. Items included standardized measures (i.e., formal clinician ratings of child/family symptoms and functioning; formal mental status exam; formal, standardized observational coding system of child/family functioning; standardized diagnostic interview; standardized symptom checklists) and unstandardized approaches (i.e., unstructured intake or clinical interview; unstructured, informal behavioral observation; and informal behavioral observations of child/family functioning). These items yielded two scores: (1) standardized measure use was the average of the five standardized measure items (α = 0.70); and (2) unstandardized measure use was the average of the three unstandardized measure items (α = 0.67). These assessment practice items were developed for the provider survey. There are no prior psychometric evaluations of these two measures, but these items have been linked to hypothesized provider- and contextual characteristics in prior studies (Cho et al., 2023; Cook et al., 2017).

#### Evidence-based Behavioral Strategies Scale (Cho et al., 2019)

The EBBSS is a 74-item measure of treatment strategies that includes EBT strategies for youth anxiety, depression, trauma, and disruptive behavior problems as well as strategies with inconsistent research support. The EBBSS was developed through a modified Delphi approach including coding of youth anxiety, depression, and disruptive behavioral EBTs for their common treatment components followed by a survey of youth EBT experts for the treatment strategies they considered core components of treatment for each of the problem areas (citation omitted). Items endorsed as core components by EBT experts were retained as strategies corresponding to EBTs while items from past practitioner surveys (Kazdin et al., 1990; Koocher & Pedulla, 1977; Silver & Silver, 1983; Tuma & Pratt, 1982; VandenBos et al., 1981), and the Psychodynamic subscale of the Therapy Procedures Checklist (TPC; Weersing et al., 2002), were included to assess treatment practices without a strong evidence base for common youth mental health concerns. Providers were asked to think about a youth with a primary diagnosis of anxiety, depression, or disruptive behavior problem (randomly assigned) with whom they had recently completed treatment and whose treatment was representative of their usual practice. Then, they were asked to rate how often they used each strategy with a recent, representative youth case (i.e., within the past year) with anxiety (*n* = 364, 33%), depression (*n* = 402, 37%), or disruptive behavior problems (*n* = 326, 30%) on a 1 (“never or almost never”) to 5 (“always or almost always”) scale. The EBBSS yields three average scores specific to the youth’s presenting concerns: (1) common EBT; (2) specific EBT; and (3) other strategies. The *common EBT* score is the average rating of items common across EBTs for youth anxiety, depression, and disruptive behavior (e.g., psychoeducation, problem-solving). The *specific EBT* score is the average rating of items specific to EBTs for the youth’s given presentation (e.g., exposure for anxiety; behavioral activation for depression; behavioral parent training for disruptive behavior). Providers’ recent, representative cases often included co-occurring problems in addition to the problem area specified in their survey version, and we accounted for comorbid problems in our scoring (e.g., the specific EBT score for a youth with anxiety and disruptive behavior included ratings for the exposure and behavioral parent training items). The *other* strategies score is the average rating of items that lack consistent research support for the youth’s presentation (e.g., interpreting dreams, play or art therapy, interpreting underlying meaning of youth’s words and actions in sessions). Providers reported the theoretical orientation of the treatment provided for this case, and all co-occurring problems.

### Data Analytic Plan

For Aim 1, we conducted a LPA to identify profiles of providers characterized by distinct patterns on five manifest dependent variables: standardized measure, unstandardized measure, common EBT, specific EBT, and other treatment strategies use. The five indicators were averages of their corresponding item ratings. While the items were rated on an ordinal scale, we treated the average scores as continuous indicators to facilitate analysis and interpretation. We sequentially tested LPA models with one to five profiles, and determined the best-fitting solution based on Akaike’s Information Criterion (AIC); Bayesian Information Criterion (BIC); sample-adjusted BIC (SABIC); Lo, Mendell, and Rubin (LMR) test; and the bootstrap likelihood ratio test (BLRT). Lower values for AIC, BIC, and SABIC are generally indicative of greater fit, but the magnitude of the decrease between the given model and the model with one less profile needs to be considered (Ferguson et al., 2020). The LMR test compares the likelihood ratios of the given model with the model with one less profile, and significant tests indicate that the given model improves fit over the more parsimonious model. BLRT similarly compares the given model and the more parsimonious model, with significant tests supporting the given model. Entropy is a measure of profile classification uncertainty, with values of 0.80 and higher indicative of “good” classification or “minimal” uncertainty of individuals into profiles (Celeux & Soromenho, 1996; Clark & Muthén, 2009; Tein et al., 2013). While not a primary model retention index, entropy can support model retention decisions (Ferguson et al., 2020) or facilitate evaluation of the extent to which profiles are differentiated in the selected model (Nylund-Gibson & Choi, 2018). The assumption of local independence was violated, so we modeled correlations between unstandardized measure use, common EBT, specific EBT, other in our LPA models (Asparouhov & Muthén, 2014; Visser & Depaoli, 2022). Missingness was low (< 1% of cases) for all five indicator variables, so analyses were conducted using full information likelihood in Mplus 8 (Muthén & Muthén, 1998–2017).

For Aim 2, we tested whether our hypothesized predictors predicted profile membership using the Bolck, Croon, and Hagenaars (BCH) approach (Bolck et al., 2004), which is recommended over traditional LPA approaches because it allows for the uncertainty of profile assignment to be included in analyses (Ferguson et al., 2020). The BCH approach involves (1) determining the best-fitting solution without any predictors in the model; (2) specifying each participant’s probability of membership in each profile while accounting for each participant’s individual class probabilities; and (3) testing whether each predictor predicts profile membership. Hypothesized predictors included four categorical variables psychology discipline (0 = no, 1 = yes), doctoral degree (0 = no, 1 = yes), learning theory orientation (0 = no, 1 = yes), and private practice setting (0 = no, 1 = yes); and nine quantitative variables time since degree, PATIE, OSC, ASA Benefit Over Clinical Judgment, Psychometric Quality, and Practicality, percentage of low-income clients, percentage of racial and ethnic minority clients, and number of comorbid diagnoses for recent, representative client. To test whether each variable predicted profile membership, we ran univariate regression models for each predictor. Then, to test their relative importance, we included all significant predictors in a single multiple regression model. LPA requires complete data for all predictors. While missingness was low for most covariates (0–7%), theoretical orientation was missing for 22.89% of providers. Data were assumed missing at random (Little & Rubin, 2019), and we used multiple imputation to generate 50 datasets for regression analyses.

For Aim 3, we report the proportion of providers who endorsed each of the top three influences. We then examined between-profile differences in rates of endorsement of the six top influences on current practice. We used the BCH approach to account for uncertainty in profile membership on these six binary distal outcomes (Asparouhov & Muthen, 2021).

## Results

### Latent Profiles of EBA and EBT Use

Table 1 includes fit indices for LPA models with up to five solutions. Fit indices did not converge on a single model, and showed that the three-, four-, and five-profile models were plausible. AIC, BIC, and SABIC supported the five-profile solution which had the lowest values for all three information criteria, but the magnitude of the decrease plateaued after three profiles. As shown in prior research with large sample sizes (e.g., Morin & Marsh, 2015; Ryan et al., 2023), the BLRT was significant for all solutions and favored the solution with *k* profiles over *k*−1. LMR clearly supported one model. LMR was significant for only the four-profile model, suggesting that the four-profile model significantly improved fit over a three-profile model. There was no significant difference in fit between the five-profile and four-profile solutions, providing support for the more parsimonious four-profile solution. Based on these indices, we selected the four-profile solution. Entropy for this solution suggested that it had the least classification uncertainty of the five solutions. Notably, the four-profile model included a small profile (4.95%) that could have been spurious (Ferguson et al., 2020; Nylund-Gibson & Choi, 2018), but this profile was consistent with practices reported in prior literature and thus retained.

**Table 1:** Summary of LPA Fit Indices for Solutions with 1-5 Profiles

Profiles	AIC	BIC	SABIC	Entropy	Classes with < 5%	Smallest class %	LMR *p*	BLRT *p*
1	11110.85	11160.81	11129.05					
2	10084.17	10164.10	10113.28	0.750	0	39%	< 0.001	< 0.001
3	9153.54	9293.42	9204.49	0.725	0	7%	0.010	< 0.001
4	9067.08	9236.94	9128.95	0.764	1	5%	0.036	< 0.001
5	9022.63	9222.46	9095.41	0.755	2	4%	0.191	< 0.001

The LMR and BLRT test if there is a difference in fit between *k* and *k*−1 classes. A significant *p*-value indicates significant improvement in fit for the solution with *k* profiles compared to the solution with *k*−1 profiles.

**Fig. 1:**
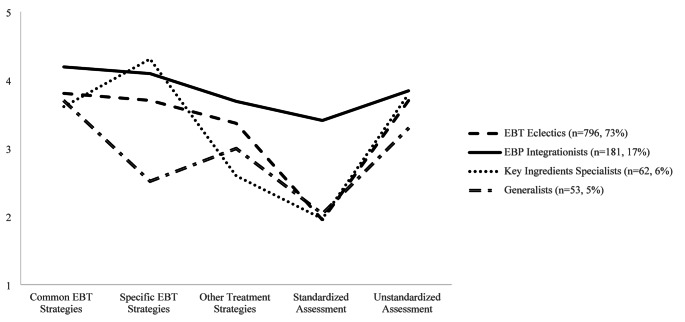
Four-Profile Solution of Assessment and Treatment Strategy Use. Rating scale 1= “never or almost never”, 2 = “rarely”, 3 = “sometimes”, 4 = “often”, 5 = “all or most of the time.” The five indicators represent the average score across included items

Table 2; Fig. 1 provide a summary of the four profiles. The largest profile (*n* = 796, 72.80%) represented “EBT Eclectics” who “sometimes” to “often” used common EBT (*M* = 3.80), specific EBT (*M* = 3.70), other treatment strategies (*M* = 3.36), and informal assessment (*M* = 3.70), but “never or almost never” to “rarely” used standardized measures (*M* = 1.95). This profile was distinguished by their relatively consistent mix of all treatment and assessment strategies except for standardized assessment. The second largest profile (*n* = 181, 16.58%) represented “EBP Integrationists” who “often” to “all or most of the time” used common (*M* = 4.19) and specific (4.09) EBT strategies, and “sometimes” to “often” used other strategies (3.69), standardized measures (*M* = 3.41), and unstandardized measures (*M* = 3.84). This profile was distinguished by their more frequent use of standardized measures compared to other profiles, and focus on common and specific EBT strategies over other treatment strategies. The third largest profile (*n* = 62, 5.68%) represented “Key Ingredients Specialists” who “often” to “all or most of the time” used specific EBT strategies (*M* = 4.31), “sometimes” to “often” used common EBT strategies (*M* = 3.61) and unstandardized measures (*M* = 3.79), “rarely” to “sometimes” used other treatment strategies (*M* = 2.59), and “never or almost never” to “rarely” used standardized assessment (*M* = 1.97). This profile had the highest specific EBT strategies rating, lowest other treatment strategies rating, and the greatest difference between these two treatment strategy types across profiles. The last profile (*n* = 53, 4.85%) represented “Generalists” who “sometimes” to “often” used common EBT (*M* = 3.70) and informal assessment (*M* = 3.29), and “rarely” to “sometimes” used specific EBT (*M* = 2.52), other treatment strategies (*M* = 3.00), and standardized measures (*M* = 2.04). This profile was distinguished by their relatively infrequent use of most treatment and assessment strategies included in the EBBSS, and were the only group to report using other treatment strategies more frequently than specific EBT strategies.

**Table 2:** Means and Standard Errors of the Indicators for Four-Profile Solution

Profile	Common EBT	Specific EBT	Other Treatment	Standardized Assessment	Informal Assessment
Generalist (*n* = 53, 5%)	3.70 (0.11)	2.52 (0.18)	3.00 (0.09)	2.04 (0.13)	3.29 (0.24)
EBP Integrationist (*n* = 181, 17%)	4.19 (0.05)	4.09 (0.06)	3.69 (0.06)	3.41 (0.11)	3.84 (0.07)
EBT Eclectic (*n* = 796, 73%)	3.80 (0.03)	3.70 (0.04)	3.36 (0.03)	1.95 (0.05)	3.70 (0.02)
Key Ingredients Specialist (*n* = 62, 6%)	3.61 (0.08)	4.31 (0.13)	2.59 (0.08)	1.97 (0.10)	3.79 (0.10)

Items were rated on a 1–5 scale where 1 = never or almost never, 2 = rarely, 3 = sometimes, 4 = often, 5 = all or most of the time. The five indicators represent the average rating across included items

### Predictors of Latent Profiles

We first tested whether each hypothesized provider and practice setting characteristic predicted profile membership in a series of univariate regression models (see Table 3). We then examined significant predictors in a multiple regression for each profile. We used the “EBP Integrationist” profile as the reference group to identify factors that distinguished the profile most similar to EBP (i.e., high frequency of both EBT strategy types and standardized measure use) from other profiles. In univariate regressions, individual provider training characteristics and attitudes differentiated EBT Eclectics and Key Ingredients Specialists from the EBP Integrationists. Compared to the EBP Integrationists, Key Ingredients Specialists were more likely to have doctoral degrees (OR = 8.28, *p* =.001) and be psychologists (OR = 5.98, *p*<.001), while EBT Eclectics were less likely to endorse learning theory orientation (OR = 0.44, *p*=.001). In terms of attitudes, EBT Eclectics (OR = 0.30, *p* <.001) and Key Ingredients Specialists (OR = 0.23, *p*<.001) reported lower PATIE scores, and EBT Eclectics reported lower ASA Benefit Over Clinical Judgment (OR = 0.39, *p* <.001), Psychometric Quality (OR = 0.33, *p*<.001), and Practicality (OR = 0.21, *p* <.001) scores. Time since degree did not predict profile membership (all *p*>.05). Practice setting characteristics also differentiated EBT Eclectics and Key Ingredients Specialists from the EBP Integrationists. Compared to EBP Integrationists, EBT Eclectics (OR = 1.92, *p* =.004) and Key Ingredients Specialists (OR = 2.14, *p* =.038) were more likely to work solely in individual private practice. EBT Eclectics reported fewer opportunities for supervision and consultation (OR = 0.63, *p* =.005). EBT Eclectics and Key Ingredients Specialists had fewer low-income (OR = 0.99, *p*=.017; OR = 0.98, *p*=.005, respectively) and racial and ethnic minority youths (OR = 0.99, *p* =.001; OR = 0.98, *p* =.004, respectively) in their case mix. Key Ingredients Specialists reported on cases with less comorbidity (OR = 0.21, *p*<.001). None of the hypothesized predictors differentiated Generalists from EBP Integrationists.

**Table 3:** Summary of Univariate and Multiple Regression Comparing Profiles to “EBT Integrationists”

	Univariate Regression						Multiple Regression					
	EBT Eclectic		Key Ingredients Specialist		Generalist		EBT Eclectic		Key Ingredients Specialist		Generalist	
	*OR*	*p*	*OR*	*p*	*OR*	*p*	*OR*	*p*	*OR*	*p*	*OR*	*p*
***Clinician Characteristics***												
ASA Benefit Over Clinical Judgment	0.39	< 0.001	0.93	0.805	0.65	0.163	0.83	0.522	1.70	0.255	0.69	0.327
ASA Practicality	0.21	< 0.001	0.64	0.212	0.50	0.074	0.19	< 0.001	0.10	< 0.001	0.51	0.151
ASA Psychometric Quality	0.33	< 0.001	1.04	0.912	0.77	0.490	1.10	0.791	1.78	0.403	1.80	0.219
Doctoral degree	1.20	0.403	8.28	0.001	0.76	0.483	2.21	0.022	8.09	0.010	1.06	0.909
Learning theory orientation	0.44	0.001	0.88	0.747	1.13	0.796	0.52	0.042	0.45	0.147	1.26	0.647
PATIE	0.30	< 0.001	0.23	< 0.001	0.47	0.076	0.33	< 0.001	0.27	0.001	0.46	0.081
Psychology	0.81	0.380	5.98	< 0.001	0.59	0.231	0.89	0.767	4.61	0.020	0.73	0.563
Time since degree^a^	1.00	0.900	1.03	0.127	0.96	0.091	--	--	--	--	--	--
***Practice Setting Characteristics***												
Comorbidity	0.93	0.458	0.21	< 0.001	0.72	0.065	0.94	0.566	0.16	0.002	0.68	0.034
Low income	0.99	0.017	0.98	0.005	1.01	0.191	1.00	0.927	1.00	0.706	1.01	0.097
Minority	0.99	0.001	0.98	0.004	1.00	0.911	0.98	0.007	0.98	0.051	0.99	0.253
Opportunities for supervision and consultation	0.63	0.005	0.85	0.512	0.85	0.574	0.74	0.106	0.91	0.783	1.05	0.877
Private practice	1.92	0.004	2.14	0.038	0.71	0.399	1.38	0.332	0.59	0.389	0.81	0.640

ASA = Attitudes Toward Standardized Assessment, PATIE = Provider Attitudes Toward Innovation and Evidence. ^a^Time since degree not included in the multiple regression due to nonsignificance in univariate regression

Next, all predictors that significantly differentiated at least one of the three profiles from EBP Integrationists were entered simultaneously into the multiple regression. Training background and attitudes remained significant predictors. Compared to EBP Integrationists, Key Ingredients Specialists were more likely to be psychologists (OR = 4.61, *p* =.020). EBT Eclectics (OR = 2.21, *p* =.022; a suppressor effect) and Key Ingredients Specialists (OR = 8.09, *p* =.010) were more likely to have doctoral degrees. EBT Eclectics were less likely to endorse learning theory orientation (OR = 0.52, *p* =.042). EBT Eclectics and Key Ingredients Specialists reported lower PATIE (OR = 0.33, *p* <.001; OR = 0.27, *p* =.001, respectively) and ASA Practicality (OR = 0.19, *p* <.001; OR = 0.10, *p* <.001, respectively) scores. Most practice setting characteristics were no longer significant in the multiple regression. However, compared to EBP Integrationists, EBT Eclectics had fewer racial and ethnic minority youths in their case mix (OR = 0.98, *p* =.007), and Key Ingredients Specialists (OR = 0.16, *p* =.002) and Generalists (OR = 0.68, *p*=.034; a suppressor effect) reported on cases with less comorbidity.

### Post-Hoc Exploration of Suppression Effects

In three instances, predictors that did not differentiate a given profile from EBP Integrationists in the univariate regression, differentiated that profile from EBP Integrationists in the multiple regression. These patterns reflect suppressor effects where inclusion of covariates (i.e., suppressor variables) in multiple regression removes variance from the outcome variable and thus strengthens the relationship between the predictor and outcome variable (Cohen et al., 2013). There was substantial multicollinearity among the predictors included in the multiple regression (see Table 4). To better understand the suppressor effects, we ran a series of post-hoc regressions that included the predictor that became significant in the multiple regression and a covariate (i.e., each of the other predictors in the multiple regression model). Covariates that when added to the regression made the predictor a significant predictor were identified as suppressor variables (Pandey & Elliott, 2010).

**Table 4:** Correlation Matrix of Predictors in Multiple Regression

Predictor	1	2	3	4	5	6	7	8	9	10	11	12
(1) ASA BOCJ	1.00											
(2) ASA P	0.56^***^	1.00										
(3) ASA PQ	0.57^***^	0.56^***^	1.00									
(4) Doctoral	0.12^***^	0.28^***^	0.25^***^	1.00								
(5) Learning Theory	0.19^***^	0.13^***^	0.20^***^	0.06	1.00							
(6) PATIE	0.09^**^	0.07^*^	0.14^***^	− 0.07^*^	0.12^***^	1.00						
(7) Psychology	0.20^***^	0.35^***^	0.28^***^	0.54^***^	0.11^**^	− 0.09^**^	1.00					
(8) Comorbidity	− 0.05	− 0.09^**^	− 0.02	− 0.06	− 0.14^***^	0.07^*^	− 0.09^*^	1.00				
(9) Low-Income	0.05	− 0.08^*^	− 0.02	− 0.13^***^	− 0.04	0.04	− 0.21^***^	0.18^***^	1.00			
(10) Minority	0.03	− 0.06	0.01	0.00	− 0.01	− 0.01	− 0.09^**^	0.17^***^	0.55^***^	1.00		
(11) Opportunities for supervision and Consultation	0.06^*^	0.16^***^	0.05	0.04	− 0.04	0.05	0.04	0.01	− 0.09^**^	− 0.03	1.00	
(12) Private practice	− 0.10^**^	− 0.02	− 0.09^**^	0.08^*^	− 0.04	− 0.05	0.18^***^	− 0.11^***^	− 0.51^***^	− 0.37^***^	0.08^**^	1.00

^*^p<.05, ^**^p<.01, ^***^p<.001

First, doctoral degree was not a significant predictor of EBT Eclectic profile membership in the univariate regression. When ASA Psychometric Quality and Practicality (both positively correlated with doctoral degree, and negative predictors of Eclectic EBT profile membership) were added as covariates, doctoral degree significantly predicted greater likelihood of membership in the EBT Eclectic than EBP Integrationist profile. Second, ASA Practicality was not a significant predictor of Key Ingredients Specialist profile membership in the univariate regression. When psychology discipline and doctoral degree (both positively correlated with ASA Practicality, and positive predictors of Key Ingredients Specialist profile membership) were added as covariates, higher ASA Practicality became a predictor lower likelihood of membership in the Key Ingredients Specialist versus EBP Integrationist profile. Third, comorbidity was not a significant predictor of the Generalist profile in the univariate regression. When percentage of low-income cases (positively correlated with comorbidity, and nonsignificant predictor of the Generalist profile) and ASA Practicality (negatively correlated with comorbidity, and non-significant predictor of Generalist profile) were added as covariates, higher comorbidity predicted a lower likelihood of membership in the Generalist (versus EBP Integrationist) profile.

### Provider-Reported Influences on Current Practice

Overall, the most frequently endorsed influence on current practice was continuing education (74.29%), followed by talking with colleagues (43.87%), professional journals or books (38.62%), graduate school training (34.29%), practicum or internship or residency (33.64%), treatment manuals or practice guidelines (18.25%), other (14.56%), and insurance coverage rules (10.51%). Comparing across profiles, rates of endorsement of professional journals or books (*Χ*^*2*^(3) = 9.05, *p*=.029) differed (see Table 5). Key Ingredients Specialists were more likely to endorse professional journals or books (*Χ*^*2*^(1) = 7.29, *p*=.007) than EBT Eclectics.

**Table 5:** Comparison of Provider Self-Reported Top Three Influences on Current Practices Between Profiles

Influence on current practice	Overall	EBT Eclectics	Key Ingredients Specialists	Generalists	EBP Integrationists	X^2^	df	*p*
Continuing education	74.29%	73.20%	79.03%	78.85%	76.11%	4.16	3	0.244
Talking with colleagues	43.87%	44.63%	35.48%	40.38%	44.44%	0.92	3	0.820
Professional journals or books	38.62%	35.52%	48.39%	42.31%	47.78%	9.05	3	0.029
Graduate school	34.29%	35.78%	40.32%	30.77%	26.67%	5.50	3	0.139
Practicum, internship, or residency	33.64%	34.51%	35.48%	30.77%	30.00%	2.33	3	0.507
Treatment manuals or practice guidelines	18.25%	15.93%	14.52%	26.92%	27.22%	3.35	3	0.340
Other	14.56%	15.04%	11.29%	15.38%	13.33%	0.66	3	0.883
Insurance coverage rules	10.51%	9.86%	9.68%	11.54%	13.33%	2.00	3	0.572

## Discussion

The current study identified four distinct profiles of youth mental health providers’ treatment and assessment practices, showing that providers vary in the mix of assessment and treatment strategies they deliver to youths in routine care settings. Only the EBP Integrationists, comprising 17% of the sample, showed frequent use of both common and specific EBT strategies and standardized measures, consistent with the practice of EBP (American Psychological Association, 2006). These providers also used other treatment strategies and informal assessments, integrated alongside EBT strategies and standardized measures. The remaining 83% of providers uniformly reported infrequent standardized measure use, but varied in their use of the three treatment strategy types. EBT Eclectics, the largest profile making up 73% of providers, used all three treatment strategy types “sometimes” to “often,” suggesting a balanced eclectic mix of treatment strategies across the range of research support. Key Ingredients Specialists (6%) reported using specific EBT strategies “often” to “always or almost always”, common EBT strategies “sometimes” to “often”, and other strategies “rarely” to “sometimes”. This pattern may reflect a selective focus on treatment strategies with the strongest research support and a deemphasis on other treatment strategies. Lastly, Generalists (5%) reported using common EBT strategies “sometimes” to “often” while “rarely” to “sometimes” using other treatment and specific EBT strategies. Generalists were the only profile with higher average ratings for other treatment strategies (mean 3.00) than specific EBT strategies (mean 2.52). These providers may focus on general treatment strategies that can be used in therapy irrespective of their research evidence or the youth’s primary presenting problems.

These findings are consistent with research findings from prior studies that used LPA to independently explore patterns of assessment (Cook et al., 2017) and treatment (Becker-Haimes et al., 2019) practices. Cook et al. (2017) reported that the majority (77%) of providers primarily used unstandardized measures with limited standardized measures use. We found the same in our study, with EBT Eclectics, Key Ingredients Specialists, and Generalists (together making up 83% of the sample) reporting minimal standardized measure use and more frequent unstandardized measure use. Cook et al. (2017) also found two profiles comprising 18% of their sample that reported at least “sometimes” used standardized measures (primarily standardized checklists and diagnostic interviews), which was similar to the 17% of providers in our EBP Integrationist profile, the sole profile to report at least “sometimes” using standardized measures alongside unstandardized measures. For treatment practices, Becker-Haimes et al. (2019) distinguished providers based on their use of treatment strategies from four theoretical orientations (i.e., behavioral, cognitive, family, psychodynamic). The behavioral and cognitive strategies overlapped with our common and specific EBT strategies, while the psychodynamic strategies overlapped with our other treatment strategies. Becker-Haimes et al. (2019) reported that their largest profile, “Moderate Eclectics” (53%), reported “seldom” to “often” using treatment strategies that spanned cognitive, behavioral, and psychodynamic orientations. The largest profile in our sample, EBT Eclectics (73%), similarly reported “sometimes” to “often” using treatment strategies that spanned the evidence base continuum including strategies from cognitive, behavioral, and psychodynamic orientations. Becker-Haimes et al. (2019) also reported on one profile, “Low Eclectics” (16%), who reported limited use of all treatment strategies with a slight preference for cognitive strategies, which was similar to our small Generalist (5%) profile that “rarely” to “sometimes” used specific EBT and other treatment strategies but reported “sometimes” to “often” using common EBT strategies, which include cognitive strategies. The inclusion of both assessment and treatment strategies in the current study allowed us to explore how these assessment and treatment practices may co-occur in routine care. We found that the small group of providers who use standardized measures also use EBT strategies. In contrast, most providers reported limited use of standardized measures and applied treatment strategies that span the continuum of research evidence. Taken together, these patterns highlight the need for training to support providers to implement common and specific EBT strategies guided by standardized assessment in line with EBP.

Several provider training-related characteristics differentiated EBP Integrationists from other providers. Key Ingredients Specialists were more likely to be doctoral-level psychologists than EBP Integrationists. Psychology doctoral programs have historically emphasized training in EBTs (Weissman et al., 2006), which may have contributed to the Key Ingredients Specialists’ relatively more frequent use of specific EBT strategies and less frequent use of other treatment strategies compared to EBP Integrationists. However, this finding is inconsistent with a prior survey study that used variable-centered analyses to show that doctoral-level psychologists reported more frequent standardized measures use than non-doctoral providers from other disciplines (Palmiter Jr, 2004). Predictors of treatment and assessment practices in isolation may differ from those that predict patterns of their integration, and use of person-centered approaches could add to a fuller understanding of how providers mix a range of youth mental health services. EBT Eclectics were less likely to report learning theory orientation than EBP Integrationists, which may be a reflection of the many EBTs that are based in learning theory (Weisz et al., 2017) and recommend routine assessment (e.g., Ehrenreich-May et al., 2025). On the other hand, Key Ingredients Specialists who rarely used standardized measures and Generalists whose treatment practices were arguably the most distinct from EBP Integrationists’, did not differ from EBP Integrationists in endorsement of learning theory, and influence of treatment manuals and practice guidelines did not differ across profiles. Thus, increased exposure to these practices may not entirely explain differences in use. It will be important to clarify under what conditions, and for which providers, EBT manuals and practice guidelines enhance treatment and assessment practices.

Provider attitudes also distinguished EBP Integrationists from other providers. EBT Eclectics and Key Ingredients Specialists reported poorer attitudes toward standardized measures compared to EBP Integrationists. This general pattern is consistent with prior work showing that poorer attitudes toward standardized measures predicted less frequent use (Jensen-Doss et al. 2018a, b, 2024; Jensen-Doss & Hawley, 2010). EBT Eclectics reported poorer attitudes toward innovation and evidence than EBP Integrationists, which aligns with prior work showing that negative perceptions toward evidence are a barrier to EBT adoption (Beidas et al., 2017; Cho et al., 2019; Nelson & Steele, 2007; Okamura et al., 2019). However, it is puzzling that Key Ingredients Specialists, who reported the most frequent use of specific EBT strategies, also had poorer attitudes toward evidence and innovation. One possibility is that standardized measures may have been perceived as a more recent innovation, and thus, attitudes toward innovation and evidence were contributing to differences in standardized assessment use between these two profiles. It is also possible that attitudes toward evidence and attitudes toward innovation (regardless of actual evidence) may be worth examining separately in future research.

Practice setting characteristics also differentiated EBP Integrationists from other providers. EBT Eclectics and Key Ingredients Specialists, but not Generalists, were more likely to work solely in individual private practice than EBP Integrationists. Private practice settings are less likely to have requirements for use of specific interventions and assessments compared to agencies (Hyzak et al., 2023). EBT Eclectics and Key Ingredients Specialists’ treatment and assessment practices may reflect fewer expectations to use EBT and standardized measures in individual private practice settings. EBT Eclectics also reported fewer opportunities for supervision and consultation. Without adequate supervision and consultation to guide adherent and skillful service delivery (Beidas and Kendall 2010; Frank et al. 2020a, b; Herschell et al. 2010), EBT Eclectics may not have received the feedback necessary to emphasize specific EBT strategies and integrate them with standardized measures. All three case mix characteristics were linked with profile membership. EBT Eclectics and Key Ingredients Specialists reported fewer low-income and racial and ethnic minority youths in their case mix than EBP Integrationists. Compared to these two profiles, EBP Integrationists may have used standardized measures to guide treatment delivery given the relatively smaller evidence base for minoritized youths (Pina et al., 2019). Key Ingredients Specialists also reported on cases with less comorbidity than EBP Integrationists did. With comorbidity an often cited barrier to EBT implementation in routine care settings (Nelson et al., 2006), EBP Integrationists may have reached for more common EBT and other treatment strategies, alongside specific EBT strategies, for transdiagnostic coverage across multiple co-occurring problems for their higher comorbidity cases, while Key Ingredients Specialists may have focused on specific EBT strategies for more streamlined treatment needs of their lower comorbidity cases. It is also plausible that Key Ingredients Specialists simply missed comorbidity given their less frequent use of standardized measures (Jensen & Weisz, 2002). These case mix characteristics findings should also be considered in light of EBT Eclectics and Key Ingredients Specialists’ greater likelihood of working solely in individual private practice than EBP Integrationists. Private practitioners have low rates of insurance acceptance (Zhu et al., 2024) which may be connected to the confluence of characteristics: fewer low-income youths, fewer racial and ethnic minority youths, and lower comorbidity (Udalova et al., 2022). Thus, youths with these demographic and clinical characteristics may be less likely to receive care in private practice settings, which may have fewer expectations for EBT and standardized measures use (Frank et al., 2024; Hyzak et al., 2023).

Multiple regression results offer insights about the factors that most strongly differentiate EBP Integrationists from other providers. Psychology discipline, doctoral degree, learning theory orientation, attitudes toward innovation and evidence and standardized measures’ practicality, proportion of racial and ethnic minority cases, and comorbidity remained or became significant predictors in the multiple regression. Consistent with prior research (Jensen-Doss et al. 2018a, b), ASA Practicality was the strongest predictor to differentiate EBP Integrationists from EBT Eclectics and Key Ingredients Specialists. These latter two profiles differed more from EBP Integrationists in their assessment than treatment practices, and providers’ perceptions about the practicality of standardized measures may have driven this difference. The only factor that differentiated Generalists from EBP Integrationists was Generalists’ lower comorbidity cases. This finding runs counter to prior research indicating client comorbidity is a barrier to EBT use (Addis et al., 1999; Nelson et al., 2006). It may be that low comorbidity cases tend to receive care in settings where Generalists provide services or seek the types of treatment provided by Generalists. It was surprising that only comorbidity differentiated Generalists from EBP Integrationists given that these two profiles demonstrated the most distinct practice patterns. Future research should explore provider (e.g., training in standardized measures or EBTs) and practice setting (e.g., implementation climate) characteristics and other domains (e.g., family, broader mental health care environment; Damschroder et al., 2009) not included in this study.

Lastly, two unexpected suppression effects suggest a complex relationship between provider training and attitudes on the one hand, and practice patterns on the other. Psychology discipline, doctoral-level training and positive attitudes toward standardized measures were correlated, and once they were considered together in a multivariate model, the nature of each one’s relationship with profile membership changed. While doctoral psychology training programs have emphasized EBT training relative to other disciplines (Weissman et al., 2006), there is variability in whether and which practices are taught (Eubanks Fleming et al., 2025). When doctoral psychology programs provide EBT and standardized measure training, these trainees may develop positive attitudes, which then contribute to post-graduate practices consistent with EBP. Of concern is the finding that once attitudes are accounted for, doctoral and psychology training predicted membership in non-EBP Integrationist profiles. Again, exposure to EBTs and standardized measures in graduate programs could be contributors, but additional work is needed to understand what aspects of training influence practice patterns and how.

There were several study limitations. Providers self-reported their assessment and treatment practices. Studies have shown that providers tend to overreport their EBT use compared to observational coding (Brookman-Frazee et al., 2021; McLeod et al., 2023). While we aimed to limit social desirability bias by asking providers to report on a range of assessment and treatment practices with and without robust research support, and by using items that briefly described each practice in everyday terms without reference to the item’s evidence base, it is possible that providers overestimated their standardized measures and EBT use. Second, as with most survey studies, there may have been selection bias. Namely, providers who completed the surveys may have had more positive attitudes toward research than providers who did not respond, a characteristic which was linked to profile membership. We also sampled providers from professional guilds which may have afforded our participants greater exposure and access to standardized measures and EBTs than providers who are not part of professional organizations. Continued work is needed to explore practice patterns in a large, representative sample of youth mental health providers in routine care settings. Such work may also yield larger profiles, and increased power to detect predictors of profile membership. We observed fewer significant predictors for the two smaller profiles (5–6% of the full sample) despite their practice patterns being more distinct from EBP Integrationists than the EBT Eclectic profile. The reduced sample size for the two smaller profiles may have limited power, and replication is needed in larger samples. Third, our treatment and assessment practice items had different stems. We asked providers to report on their assessment practices with youths 3–17 years old, while we asked them to report on their treatment practices for a recent, representative case. As such, providers’ reported assessment practices may not reflect the assessment tools used with the particular case for whom treatment practices were reported. Relatedly, while providers reported the assessment tools they used for cases presenting for treatment, our survey did not explicitly ask whether and how providers integrated standardized measures with their treatment practices. It is possible that providers who administered standardized measures during treatment did not use them to guide treatment delivery (Garland et al., 2003). Additional research is needed to understand whether, how, and under what conditions standardized measures and EBTs are integrated in routine care. Importantly, such research should explore aspects of EBP not included in this study, including integration of client characteristics, culture and preferences, as well as other competencies within providers’ clinical expertise. Lastly, there were several limitations related to our measurement model. Some measures were developed for this study (e.g., OSC) or evidenced limited internal consistency (i.e., PATIE, informal measures use). While most of our outcome measures demonstrated adequate internal consistency and have demonstrated relationships with provider and contextual variables consistent with theory and empirical findings (Cho et al., 2019, 2023; Cook et al., 2017), they require further psychometric evaluation (e.g., predictive validity, convergent and discriminant validity). Lastly, we created continuous indicator variables for our assessment and treatment practices variables from ordinal items, and it is possible that these ordinal variables may not meet the assumption of normality within profiles. Replication of our findings with well-established measures is needed.

Despite these limitations, our findings shed light on some positive pasterns consistent with EBP that may not be apparent from general trends described by variable-centered approaches, lending support for use of person-centered approaches to complement traditional variable-centered approaches. Our findings highlight possible future directions to enhance EBP in routine care settings. First, while most providers reported using EBT strategies to some degree, less than a fifth reported assessment and treatment practices reflective of the EBP approach. Without routine standardized assessment to guide EBT delivery (American Psychological Association, 2006; Southam-Gerow & Prinstein, 2014), youths receiving EBTs in routine care settings may not experience the full effects shown in prior research. Providers may benefit from trainings and resources that help them to use standardized measures to guide EBT delivery. Initial training and subsequent consultation in the integration of EBTs and standardized measures has been linked to pre-to-post improvements on attitudes, skills, and use of standardized measures (Lyon et al., 2015). Such training and support could be particularly helpful for providers who may have limited access to such infrastructure (e.g., individual private practice) and desire more opportunities for supervision and consultation. Most providers (74%) endorsed continuing education as a top influence on current practice with similar rates of endorsement across profiles, suggesting that continuing education could be explored as an avenue to make EBP training accessible. A review of continuing education in healthcare showed that continuing education often in the form of workshops, courses, and seminars have small impacts on provider behaviors overall, but that outcomes vary and it remains unclear what characteristics of continuing education produce more favorable outcomes (Forsetlund et al., 2021). Specific to mental health providers, use of active learning strategies such as modeling, role-play, and feedback has been linked to provider behavior change (Bearman et al., 2017; Beidas & Kendall, 2010). Designing continuing education opportunities that incorporate active learning strategies while remaining low-burden (i.e., financial and time cost) may improve access to effective EBP training for providers in private practice and other settings with limited resources to support ongoing training. Second, of the three treatment strategy types, profiles varied most in their use of specific EBT strategies (“rarely” to “often”). Growing research demonstrating the importance of specific EBT strategies to treatment outcomes (e.g., Whiteside et al., 2020) points to the need for training approaches that improve use of these “key ingredients.” Trainings could be tailored to the factors that limit specific EBT strategies use. For example, experiential learning training approaches have been designed to reduce negative perceptions about and discomfort with exposure, a key ingredient of EBTs for childhood anxiety disorders (Frank et al. 2020a, b). Third, there may be utility to tailoring implementation strategies based on providers’ practice patterns (Becker-Haimes et al., 2019). For example, Key Ingredients Specialists demonstrated comparable use of EBT strategies to EBP Integrationists but lower standardized measures use, so training could focus on improving their poor perceptions about the practicality of standardized measures. In contrast, EBT Eclectics demonstrated minimal standardized measure use alongside relatively infrequent use of specific EBT strategies. Therefore, their training could cover standardized measures’ clinical utility, validity, and practicality (all lower than EBP Integrationists’ ASA scores), while additional supervision and consultation (lower than EBP integrationists’ OSC scores) could target specific EBT strategies use and facilitate integration of standardized measures with EBT strategies.

In conclusion, this study provided a first glimpse into patterns of individual providers’ assessment and treatment practices for common referral concerns in routine care settings. Findings show that providers engage in distinct treatment and assessment practices, with most providers using a range of treatment strategies that span the continuum of research evidence and with minimal use of standardized measures. Strategies that support providers to integrate standardized measures with EBT delivery may enhance EBP delivery in routine care settings.

## Data Availability

Data are not available as participants did not consent to data sharing at the time of consent. Materials and code are available from the corresponding author upon request.

## Declarations

### Standards of reporting

This manuscript was prepared in concordance with the STROBE guidelines for observational research.
